# Clinical Feasibility of Monitoring Resting Heart Rate Using a Wearable Activity Tracker in Patients With Thyrotoxicosis: Prospective Longitudinal Observational Study

**DOI:** 10.2196/mhealth.9884

**Published:** 2018-07-13

**Authors:** Jie-Eun Lee, Dong Hwa Lee, Tae Jung Oh, Kyoung Min Kim, Sung Hee Choi, Soo Lim, Young Joo Park, Do Joon Park, Hak Chul Jang, Jae Hoon Moon

**Affiliations:** ^1^ Department of Internal Medicine Seoul National University Healthcare System Gangnam Center Seoul Republic Of Korea; ^2^ Department of Internal Medicine Seoul National University Bundang Hospital Sungnam-si Republic Of Korea; ^3^ Department of Internal Medicine Seoul National University Hospital Seoul Republic Of Korea

**Keywords:** activity tracker, wearable device, heart rate, thyrotoxicosis, hyperthyroidism, Graves’ disease

## Abstract

**Background:**

Symptoms and signs of thyrotoxicosis are nonspecific and assessing its clinical status is difficult with conventional physical examinations and history taking. Increased heart rate (HR) is one of the easiest signs to quantify this, and current wearable devices can monitor HR.

**Objective:**

We assessed the association between thyroid function and resting HR measured by a wearable activity tracker (WD-rHR) and evaluated the clinical feasibility of using this method in patients with thyrotoxicosis.

**Methods:**

Thirty patients with thyrotoxicosis and 10 controls were included in the study. Participants were instructed to use the wearable activity tracker during the study period so that activity and HR data could be collected. The primary study outcomes were verification of changes in WD-rHR during thyrotoxicosis treatment and associations between WD-rHR and thyroid function. Linear and logistic model generalized estimating equation analyses were performed and the results were compared to conventionally obtained resting HR during clinic visits (on-site resting HR) and the Hyperthyroidism Symptom Scale.

**Results:**

WD-rHR was higher in thyrotoxic patients than in the control groups and decreased in association with improvement of thyrotoxicosis. A one standard deviation–increase of WD-rHR of about 11 beats per minute (bpm) was associated with the increase of serum free T4 levels (beta=.492, 95% CI 0.367-0.616, *P*<.001) and thyrotoxicosis risk (odds ratio [OR] 3.840, 95% CI 2.113-6.978, *P*<.001). Although the Hyperthyroidism Symptom Scale showed similar results with WD-rHR, a 1 SD-increase of on-site rHR (about 16 beats per minute) showed a relatively lower beta and OR (beta=.396, 95% CI 0.204-0.588, *P*<.001; OR 2.114, 95% CI 1.365-3.273, *P*<.001) compared with WD-rHR.

**Conclusions:**

Heart rate data measured by a wearable device showed reasonable predictability of thyroid function. This simple, easy-to-measure parameter is clinically feasible and has the potential to manage thyroid dysfunction.

**Trial Registration:**

ClinicalTrials.gov NCT03009357; https://clinicaltrials.gov/ct2/show/NCT03009357 (Archived by WebCite at http://www.webcitation.org/70h55Llyg)

## Introduction

Thyrotoxicosis is a clinical syndrome resulting from the high concentration of free thyroxine (T4) and free triiodothyronine (T3). The prevalence of thyrotoxicosis is approximately 2%, and its most common cause (ie, 60%-90%) is Graves’ disease (GD) which is an autoimmune disease that stimulates the thyroid gland to produce and release thyroid hormone [1-3]. Thyrotoxicosis results in various symptoms and signs, including fatigue, anxiety, palpitations, sweating, heat intolerance, disturbed sleep, and weight loss because the thyroid hormone affects many different organs [4]. These clinical manifestations are relatively nonspecific and vary depending on factors such as patient age and sex, disease duration, and etiology [5,6]. Although palpitation and increased heart rate (HR) are among the most frequent symptoms and signs [7], and the easiest to quantify, HR can easily be affected by other factors such as the patient’s emotional state, body position, and physical activities. Therefore, it is difficult to assess disease status based solely on patient HR that is either self-reported or obtained conventionally in the clinic during a specific moment. Although researchers have developed assessment tools that use parameters associated with symptoms and signs of thyrotoxicosis, most are questionnaire-based scoring systems that require time and educated scorers [8,9]. Thyrotoxicosis is diagnosed when a thyroid function test (TFT) shows increased serum thyroid hormone levels. This test is often delayed because patients tend to visit the clinic in severe status due to the nonspecific symptoms of thyrotoxicosis. Therefore, a simple, easy-to-measure parameter to predict thyroid status would be useful for thyrotoxicosis management.

The popularity of wearable activity trackers has grown considerably in recent years. The American College of Sports Medicine survey of fitness trends reported that wearable technology was the top-rated trend in 2016 [10]. Forecasts indicate that wearable activity tracker sales will exceed 82 million by the end of 2019, 2.5 times the expected sales volume in 2016 [11]. These devices are typically worn on the wrist or hip and provide the user with information about their physical activity, such as steps taken, vertical and horizontal moving distance, and sleep patterns. Recently developed devices can measure HR using photoplethysmography, which measures a differential reflection of light from the skin, based on the pulsatility of superficial blood vessels [12]. Many studies on the accuracy of these wrist-worn HR monitors have been published, showing relatively accurate HR during the resting state [13,14]. It is easy to collect detailed longitudinal HR data and physical activity with these wearable devices, and these data can provide more information than intermittently measured HR. This makes it now possible to collect and analyze more detailed and precise HR data, which is a crucial parameter changed in thyrotoxicosis. However, few trials have evaluated wearable devices for use in assessing thyroid dysfunction.

Therefore, the objectives of the present study were to investigate whether and how well HR data collected by commercially available activity trackers reflect thyroid function across the clinical course of thyrotoxicosis. Using this approach, the clinical feasibility of wearable devices for the management of thyroid dysfunction was evaluated.

## Methods

### Study Design and Participants

This was a single-center prospective observational study. Subjects were recruited from the outpatient clinic of the endocrinology department at Seoul National University Bundang Hospital (SNUBH).

For the thyrotoxicosis group, patients 15-60 years of age who had been diagnosed with newly developed or recurrent thyrotoxicosis were eligible to participate. Participants needed to own a mobile phone and to be able to use a wearable device and its mobile application. Among those for whom the etiology of thyrotoxicosis was GD, only patients who had planned treatment with antithyroid drugs (ATD) were included so that their clinical course could be followed during medical treatment. We prescribed methimazole as the first choice ATD unless the subjects were contraindicated. No subject had a contraindication for methimazole or adverse events during the administration of methimazole. Inclusion and exclusion criteria are listed in Multimedia Appendix 1. Prescribing beta blockers is a standard treatment for relief of thyrotoxicosis symptoms; we prescribed short-acting beta blocker propranolol to symptomatic patients to minimize its impact on the study. A total of 37 patients were screened for participation. A total of 30 patients were finally enrolled, and 2 withdrew during the study. One participant withdrew because of a skin reaction to the device (ie, Fitbit Charge HR) on their wrist; the investigators withdrew the second because of poor adherence to using the device. There were 25 and 3 patients diagnosed with GD and thyroiditis for the etiology of thyrotoxicosis, respectively, who completed the study.

Healthy adults without a history of thyroid disease were included in the control group. Consistent with the thyrotoxicosis group, participants needed to be able to use a wearable device and the mobile application. These participants were screened to ensure they were not taking medications affecting HR, including beta blockers. There were 13 potential participants screened, and 10 were enrolled in the control group. All participants were informed about the study and provided written informed consent. This study was approved by the SNUBH Institutional Review Board (IRB #B-1609-363-004) and registered on ClinicalTrials.gov (trial registration #NCT03009357).

### Procedures

**Figure 1:**
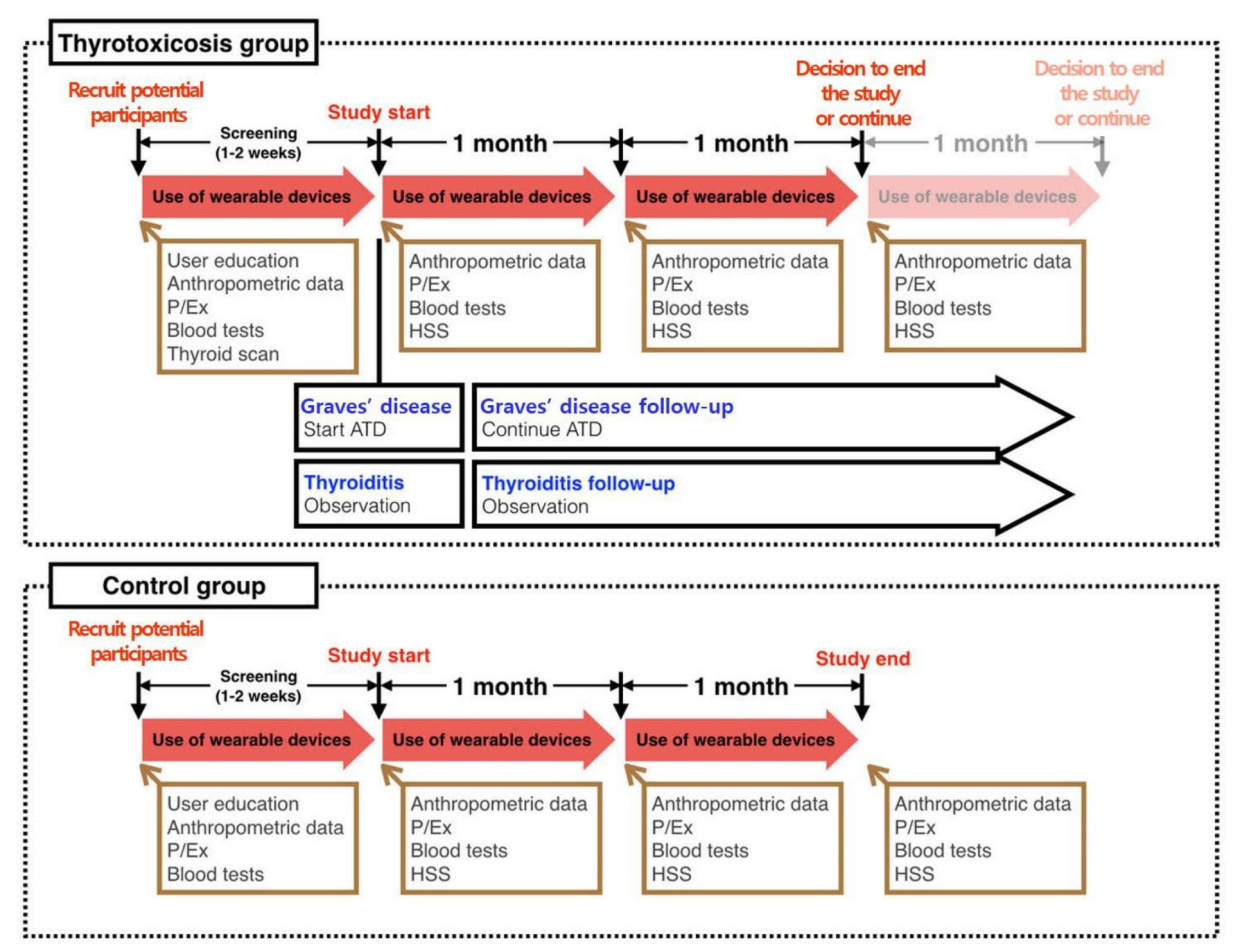
Study design and flow. Blood tests included a thyroid function test, serum levels of antithyroid-stimulating hormone receptor antibody, and other biochemical tests. Tc-99m is used in the thyroid scan. ATD: antithyroid drug, HSS: Hyperthyroidism Symptom Scale, P/Ex: physical examination.

The study design is shown in Figure 1. On the first visit, potential candidates were provided with a device and brief on-site instructions for its use. They were instructed to wear the device as much as possible throughout the day and during sleep. We also explained that if they did not wear the device or synchronize it with the application for more than five consecutive days, they could be withdrawn from the study due to poor adherence.

After a one- to two-week screening period, patients visited the clinic to confirm the results of the TFT and other tests to determine the etiology (eg, autoantibodies, thyroid scan) and to start appropriate treatment. At this visit, those who met the inclusion criteria were enrolled. Patients with GD were prescribed a specific ATD dose, as determined by the endocrinologist. Patients with thyrotoxicosis caused by thyroiditis were reassured that their symptoms and signs were benign and self-limited. Patients taking propranolol were instructed to take the medication when their symptoms were severe and to inform the investigator of their dosing times, which were recorded in the case report form (CRF). Regardless of the etiology of thyrotoxicosis, all patients had monthly follow-ups, during which time they underwent blood tests, including TFT. Their ATD dose was adjusted as necessary. The study ended after each patient’s third visit; however, the study duration could be extended at the discretion of the investigator with the patient’s consent if their TFT was not fully restored. At each visit, anthropometric data were collected, and vital signs were measured.

Healthy adults were recruited into the control group through an official SNUBH announcement. Control participants visited the hospital on the same schedule as the thyrotoxic patients. They were given the same instruction about using the device and mobile application, and they were also instructed to wear the device all day and to inform to the investigator of any medication changes, which were recorded in the CRF. Control participants’ visit schedules and blood tests were consistent with those of thyrotoxic patients, but the duration of their study participation was not extended.

### Wearable Devices and Applications

We used the Fitbit Charge HR or Fitbit Charge 2 (Fitbit, San Francisco, CA) and the Fitbit application for iOS (Apple, Cupertino, CA) or Android (Google, Mountain View, CA). The firmware versions of these devices were 18.128 for Fitbit Charge HR and 22.53.4 for Fitbit Charge 2 at the end of the study, and the latest version was maintained continuously over the study period. Although we started the study with Fitbit Charge HR, when Fitbit released Fitbit Charge 2, they discontinued production of the former device. Therefore, we used Fitbit Charge 2 with study participants enrolled after March 2017 (n=7 in the control group). However, these 2 models share a common sensor and data processing algorithm for both activity tracking and HR measurement. Activity and HR data are collected by the 3-axis accelerometer and plethysmography sensor, respectively. These sensors are equipped with the device. During the study period, each participant’s Fitbit account information, including identification and password, were shared with researchers, allowing us to access their online Fitbit account [15] to monitor their synchronization status between the Fitbit data server and tracking device. After the end of their participation, we separated the account from the device, and each participant retained their account.

### Hyperthyroidism Symptom Scale

To assess their hyperthyroidism clinical status at each clinic visit, the endocrinologist overseeing this study evaluated the patients using the Hyperthyroidism Symptom Scale (HSS) [16,17]. The HSS consists of 10 items, including 1 psychometric item (ie, nervousness) and 9 items about physical response in the thyrotoxic state. A total of 8 items were evaluated through history taking: nervousness, sweating, heat intolerance, hyperactivity, weakness, diarrhea, appetite, and assessment of daily function. Physical examination evaluated the remaining 2 items: tremor and hyperdynamic precordium. Item scores were totaled to obtain an overall HSS score, which could range from 0-40 points.

### Definition of Resting Heart Rate

The American Heart Association defines the resting heart rate (rHR) as the heart beats per minute (bpm) pumping the lowest amount of blood someone needs when they are in a resting position. The rHR can be changed by emotional state, medication, or current disease. In this study, we focused on rHR because we expected thyrotoxicosis to affect rHR significantly. We used 3 different rHR parameters according to the measurement method and calculating algorithm as follows. On-site rHR is the heart rate measured manually on the right wrist (ie, radial pulse) in a seated position after at least 10 minutes of resting. To calculate rHR from the HR log generated by the wearable device, we downloaded daily summary and detailed HR and activity data from the Fitbit database in JavaScript object notation (JSON) format using the application programming interface provided by Fitbit [18]. The process of data extraction to JSON has been described in detail in a previous paper [19]. From the HR log collected each day, we extracted HR data within time windows based on the absence of physical activity during the preceding 15 or more minutes and designated the median value of the extracted HR as the rHR of each day. In this study, rHR measured by a wearable device was defined as the mean 5-day rHR value extracted from the HR log collected by the wearable device before each TFT visit. The term WD-rHR-own refers to rHR data calculated by the above-described algorithm. Fitbit has an algorithm for generating rHR data from the HR and activity logs obtained from the tracking device. In a similar way, the term WD-rHR-Fitbit refers to rHR data based on the Fitbit algorithm. On-site rHR is consistent with the conventional definition of rHR, but the concept of WD-rHR does not guarantee that the participant is in a resting position during the measurement. This is because a lack of physical activity as detected by the device does not necessarily mean they are in a resting position. Although the exclusion criteria could control medication and other diseases, the emotional state was not controlled or evaluated in this study.

### Anthropometric and Biochemical Measurements

We measured the subjects’ height and weight while wearing light clothing and without shoes to the nearest 0.1 cm and 0.1 kg, respectively. Body mass index was calculated by determining the ratio between weight and the square of the height and expressed in kilograms per square meter. Right arm blood pressure was measured with the subject in a seated position after at least 10 minutes of resting. Biochemical measurements including TFT are listed in the Multimedia Appendix 1. The free T4 assay had an analytic sensitivity of 0.05 nanograms per deciliter (ng/dL), and TSH had an analytical sensitivity of 0.04 milli-international units per liter (mIU/L) and functional sensitivity of 0.07 mIU/L. The reference ranges for free T4 and TSH were 0.89-1.79 ng/dl and 0.3-4.0 mIU/L, respectively. Thyrotoxicosis was defined based on the results of the TFT (ie, overt thyrotoxicosis was defined as higher free T4 and lower TSH levels than respective reference ranges; subclinical thyrotoxicosis was defined as normal free T4 and lower TSH levels). All subjects were examined for the presence of anti-TSH receptor antibody by radioimmunoassay (Cis Bio International), and the cutoff for positivity was greater than 1.0 units per milliliter (U/mL).

### Data Analysis

Data were expressed as the mean (SD) or median (interquartile range). To compare variables between the patient and control groups, we used Student *t* test or the Mann Whitney *U* test for continuous variables and the chi-square test or Fisher exact probability test for categorical variables. Repeated measures analysis of variance (ANOVA) or Friedman test were used to analyze changes in thyroid hormone levels and associated parameters (HSS, on-site rHR, and WD-rHR) during the study period. The relationship between thyroid hormone levels and each associated parameter was determined by linear model generalized estimating equation (GEE) analyses. The relationship between thyrotoxicosis, defined as 1.8 ng/dL or more of free T4, and each associated parameter was assessed using binary logistic model GEE analyses. Odds ratios (OR) and 95% CI were computed per one standard deviation increase in these variables to the risk of thyrotoxicosis. A two-tailed *P*<.05 was considered statistically significant. All statistical analyses and data preparation were performed using IBM SPSS Statistics (version 20.0; IBM Corp, Armonk, NY, USA) and R (version 3.3.3; The R Foundation for Statistical Computing, Vienna, Austria).

## Results

### Participant Baseline Characteristics

Participant baseline characteristics are summarized in Table 1. There were no differences in age, sex ratio, or body mass index between the 28 patients with thyrotoxicosis (ie, 25 diagnosed with GD, 3 with thyroiditis) and the 10 controls. As expected, on-site rHR in the thyrotoxicosis group was significantly higher than that in the control group. Blood pressure did not differ between the groups. There was a significantly higher HSS score and free T4, and lower TSH, in the thyrotoxic patients compared with the controls. Also, there were significant differences between the 2 groups on several parameters known to be related to thyrotoxicosis, including total cholesterol, serum creatinine, platelets, and liver enzymes. The mean duration of observation was 3.27 (SD 1.21) months for all subjects, which was longer in thyrotoxic patients than in controls.

### Changes in Thyroid Function and Associated Parameters

**Figure 2:**
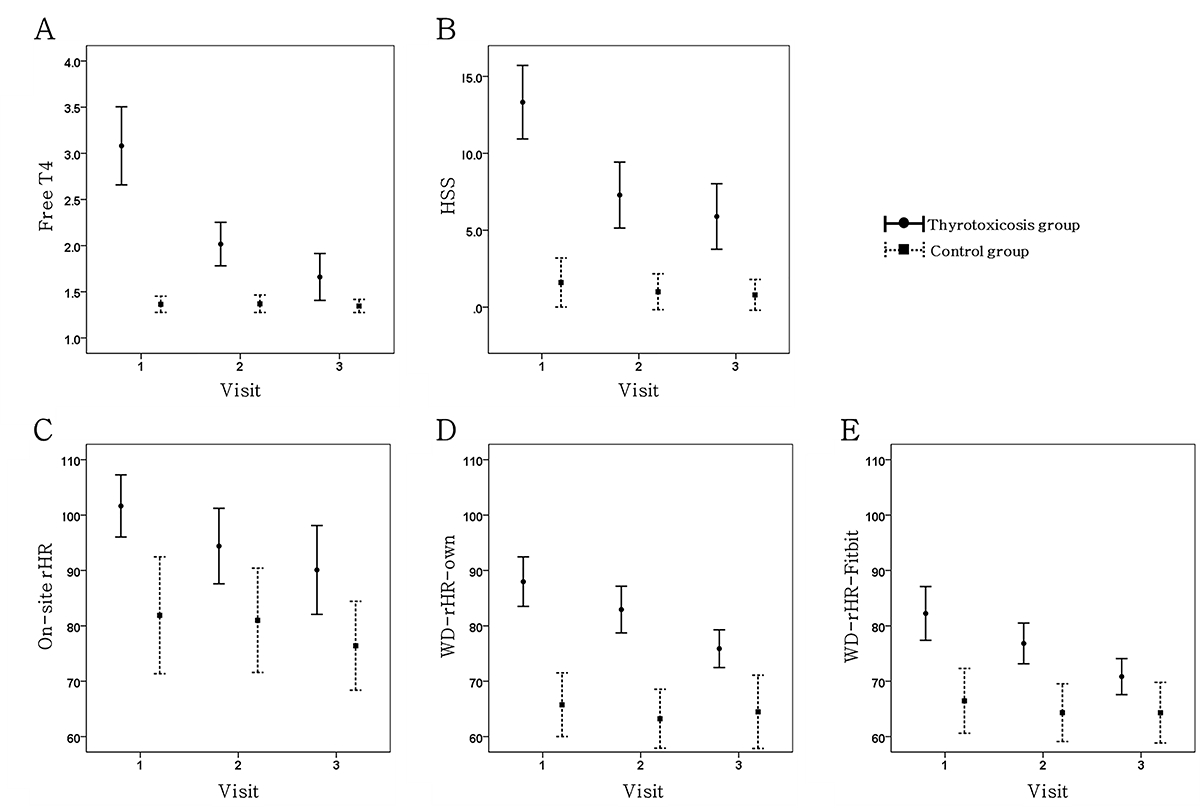
Change of serum free thyroxine (T4) levels (A), hyperthyroid symptom scale (B), on-site heart rate (C), and resting heart rate from wearable device (D) during the study period. HSS: Hyperthyroid Symptom Scale, rHR: resting heart rate, WD-rHR-own: rHR from wearable device derived by own algorithm, WD-rHR-Fitbit: derived by Fitbit algorithm. Error bars represent 95% CI of the means.

In thyrotoxic patients, thyrotoxicosis improved with treatment, as shown by decreased serum free T4 levels through the third visit. Serum TSH levels remained unchanged during the observation period (Table 2). Thyroid function was not fully restored in all patients by the end of the study, at which time 6 patients improved to a euthyroid state, 17 remained sub-clinically thyrotoxic, and 5 were still overtly thyrotoxic (data not shown). HSS, on-site rHR, and WD-rHRs also decreased with treatment in the thyrotoxicosis group through the third visit (Table 2 and Figure 2). This study was scheduled to terminate after the third visit but was extended, with patient consent, in those whose thyroid function was not restored. Thus, increased free T4, HSS, and WD-rHRs were apparent after the fourth visit because only patients whose thyroid function had not yet recovered were retained in the study through a fifth visit. The control group completed the study at the third visit, and none of their thyroid hormone levels or associated parameters differed between visits.

**Table 1:** Baseline characteristics.

Characteristics		Thyrotoxicosis (n=28)	Control (n=10)	*P* value
Age (years), mean (SD)		34.9 (10.9)	34.1 (5.9)	.78^a^
**Gender, n (%)**				
	Male	10 (36)	3 (30)	.53^b^
	Female	18 (64)	7 (70)	
Body mass index (kg/m^2^), mean (SD)		20.6 (4.7)	20.7 (1.6)	.93^a^
**Blood pressure (mmHg), mean (SD)**				
	Systolic blood pressure	130.2 (14.3)	124.6 (11.4)	.27^a^
	Diastolic blood pressure	78.1 (10.6)	70.6 (9.6)	.06^a^
On-site resting heart rate (bpm^c^), mean (SD)		101.6 (14.5)	81.9 (14.8)	.001^a^
Hyperthyroid symptom scale, mean (SD)		12.5 (10.0)	0.5 (2.8)	<.001^d^
**Thyroid function test**				
	Free thyroxine (ng/dL), mean (SD)	3.08 (1.09)	1.36 (0.12)	<.001^a^
	Thyroid stimulating hormone (mIU/L), median (IQR^e^)	0.01 (0.00)	1.33 (1.19)	<.001^d^
Thyrotropin-binding inhibitory immunoglobulin (IU/L), median (IQR)		4.2 (10.3)	—	—
Glucose (mg/dL), mean (SD)		105.8 (18.6)	96.6 (23.1)	.24^a^
Blood urea nitrogen (mg/dL), mean (SD)		13.0 (2.9)	11.7 (2.4)	.20^a^
Creatinine (mg/dL), mean (SD)		0.50 (0.20)	0.69 (0.19)	.01^a^
Total cholesterol (mg/dL), mean (SD)		141.7 (22.1)	180.2 (22.8)	<.001^a^
Total protein (g/dL), mean (SD)		7.1 (0.5)	7.2 (0.4)	.37^a^
Albumin (g/dL), mean (SD)		4.3 (0.3)	4.4 (0.3)	.17^a^
Total bilirubin (mg/dL), mean (SD)		0.69 (0.30)	0.65 (0.26)	.69^a^
Aspartate aminotransferase (mg/dL), mean (SD)		26.4 (10.4)	18.3 (3.1)	.001^a^
Alanine aminotransferase (mg/dL), mean (SD)		33.1 (22.0)	15.1 (7.9)	.02^a^
White blood count (no/mm^3^), mean (SD)		5347.3 (1980.1)	5857.0 (1397.6)	.46^a^
Hemoglobin (mg/dL), mean (SD)		14.0 (1.44)	13.6 (1.4)	.42^a^
Platelet (no/mm^3^), mean (SD)		182,200 (118,600)	276,600 (59,100)	.02^a^

^a^Derived from Student *t* test.

^b^Derived from Fisher exact probability test.

^c^bpm: beats per minute.

^d^Derived from Mann Whitney *U* test.

^e^IQR: interquartile range.

**Table 2:** Change of thyroid function and associating parameters during the study period in thyrotoxicosis and control groups.

Thyroid function test results and associated parameters		Visit 1		Visit 2		Visit 3		Visit 4		Visit 5		*P* value^a^
**Thyrotoxicosis group, n**		28		28		28		23		5		—
	Free thyroxine (ng/dL), mean (SD)		3.08 (1.09)		2.02 (0.61)		1.66 (0.64)		1.60 (0.59)		1.96 (0.39)	<.001^b^
	Thyroid stimulating hormone (mIU/L), median (IQR^c^)		0.01 (0.00)		0.01 (0.00)		0.01 (0.13)		0.01 (0.10)		0.01 (0.10)	.114^d^
	Hyperthyroid Symptom Scale, median (IQR)		12.5 (10.0)		5.5 (7.8)		4.0 (8.8)		3.5 (4.5)		7.0 (4.5)	<.001^d^
	On-site resting heart rate (bpm^e^), mean (SD)		101.6 (14.5)		94.4 (15.4)		90.1 (17.6)		85.5 (12.0)		83.5 (4.0)	.015^b^
	WD-rHR-own^f^ (bpm), mean (SD)		88.0 (11.5)		82.9 (10.9)		75.9 (8.8)		76.2 (8.0)		72.7 (7.2)	<.001^b^
	WD-rHR-Fitbit^g^ (bpm), mean (SD)		82.2 (12.5)		76.8 (9.5)		70.8 (8.4)		71.4 (7.8)		74.7 (9.8)	<.001^b^
**Control group, n**		10		10		10		—		—		—
	Free thyroxine (ng/dL), mean (SD)		1.36 (0.12)		1.37 (0.13)		1.35 (0.10)		—		—	.285^b^
	Thyroid stimulating hormone (mIU/L), median (IQR)		1.33 (1.19)		1.62 (2.21)		2.06 (1.21)		—		—	.236^d^
	Hyperthyroid Symptom Scale, median (IQR)		0.5 (2.8)		0.0 (2.0)		0.0 (2.0)		—		—	.504^d^
	On-site resting heart rate (bpm), mean (SD)		81.9 (14.8)		81.0 (13.2)		76.4 (11.2)		—		—	.250^b^
	WD-rHR-own (bpm), mean (SD)		65.8 (8.0)		63.2 (7.4)		64.5 (9.2)		—		—	.374^b^
	WD-rHR-Fitbit (bpm), mean (SD)		66.5 (8.2)		64.3 (7.3)		64.3 (7.7)		—		—	.101^b^

^a^Visit 1 to 3 were compared both in thyrotoxicosis and control groups.

^b^Derived from ANOVA with repeated measures with a Greenhouse-Geisser correction.

^c^IQR: interquartile range.

^d^Derived from Friedman test.

^e^bpm: beats per minute.

^f^WD-rHR-own: the resting heart rate from wearable device derived by own algorithm.

^g^WD-rHR-Fitbit: the resting heart rate from wearable device derived by Fitbit algorithm.

### Association Between Serum Free Thyroxine Levels and Each Associated Parameter

Linear model GEE analyses were performed to verify the relationship between serum free T4 levels and each associated parameter (ie, HSS, on-site rHR, WD-rHR-own, and WD-rHR-Fitbit). In these analyses, the mean (SD) of each parameter were standardized to 0 and 1, respectively, to compare each parameter’s relationship with free T4 levels and with each other. Before standardization, the 1SD of HSS, on-site rHR, WD-rHR-own, and WD-rHR-Fitbit were 6.3, 15.8, 11.4, and 11.2, respectively in thyrotoxic patients and 6.3, 16.2, 11.4, and 11.4 in all study participants. Although all parameters analyzed were significantly associated with serum free T4 levels in thyrotoxicosis patients and in all study participants, unstandardized beta for serum free T4 level by an increase of 1 SD of on-site rHR was relatively lower than those of other parameters (Table 3). To control thyrotoxicosis symptoms, some patients took a beta blocker intermittently. We performed the same analysis, dividing the patient group into beta blocker users (n=13) and nonbeta blocker users (n=15), which did not change the interpretation of the results (Multimedia Appendix 1).

### Odds Ratio of Each Associated Parameter for Thyrotoxicosis

The relationship between thyrotoxicosis, defined as 1.8 ng/dL or more of free T4, and each associated parameter was assessed with binary logistic model GEE analyses. All parameters analyzed were significant for predicting thyrotoxicosis (Table 4). Also, in this analysis, OR for thyrotoxicosis by a 1 SD increase in on-site rHR was relatively lower than those of other parameters, HSS, WD-rHR-own, and WD-rHR-Fitbit, which showed similar ORs (Table 4). These findings were consistent when analyzing beta blocker users and nonusers separately, except that on-site rHR was nonsignificant for predicting thyrotoxicosis in the nonbeta blocker users (Multimedia Appendix 1).

**Table 3:** Linear model generalized estimating equations analyses for the association between free thyroxine and associating parameters. Parameters standardized to the same mean and SD (mean 0, SD 1.0) for comparison and analyzed separately.

Association between free thyroxine and parameters		Unstandardized beta	95% CI	*P* value
**Thyrotoxicosis group only**				
	Hyperthyroid Symptom Scale	.504	0.333-0.674	<.001
	On-site resting heart rate	.362	0.122-0.602	.003
	WD-rHR-own^a^	.465	0.300-0.630	<.001
	WD-rHR-Fitbit^b^	.513	0.331-0.694	<.001
**All groups**				
	Hyperthyroid Symptom Scale	.541	0.394-0.687	<.001
	On-site resting heart rate	.396	0.204-0.588	<.001
	WD-rHR-own	.492	0.367-0.616	<.001
	WD-rHR-Fitbit	.515	0.375-0.656	<.001

^a^WD-rHR-own: resting heart rate from wearable device derived by own algorithm.

^b^WD-rHR-Fitbit: resting heart rate from wearable device derived by Fitbit algorithm.

**Table 4:** Binary logistic model generalized estimating equations analyses for the association between thyrotoxicosis and associating parameters. Parameters are standardized to have same mean and SD (mean 0, SD 1.0) for comparison and analyzed separately.

Association between free thyroxine and parameters		Odds ratio	95% CI	*P* value
**Thyrotoxicosis group only**				
	Hyperthyroid Symptom Scale	2.614	1.612-4.238	<.001
	On-site resting heart rate	1.648	1.047-2.594	.031
	WD-rHR-own^a^	2.475	1.470-4.166	<.001
	WD-rHR-Fitbit^b^	2.863	1.649-4.970	<.001
**All groups**				
	Hyperthyroid Symptom Scale	3.601	2.190-5.923	<.001
	On-site resting heart rate	2.114	1.365-3.273	<.001
	WD-rHR-own	3.840	2.113-6.978	<.001
	WD-rHR-Fitbit	3.843	2.067-7.144	<.001

^a^WD-rHR-own: resting heart rate from wearable device derived by own algorithm.

^b^WD-rHR-Fitbit: resting heart rate from wearable device derived by Fitbit algorithm.

## Discussion

### Prinicipal Findings

To evaluate the clinical feasibility of wearable device generated data for the management of thyrotoxicosis, we investigated the association between HR data collected by activity trackers and thyroid function across the clinical course of thyrotoxicosis. Our results demonstrated that both rHR measured by a wearable device and HSS were significantly associated with thyroid function.

It is well known that the thyroid hormone has a positive chronotropic, inotropic effect, meaning that it stimulates the rate and force of systolic contraction and the rate of diastolic relaxation [7]. Therefore, thyrotoxicosis increases systolic blood pressure and HR and widens pulse pressure [20]. In this study, the thyrotoxicosis group showed elevated on-site rHR compared with the control group. Mean values for systolic and diastolic blood pressures were higher in the thyrotoxicosis group, but this difference was not statistically significant. Also, several parameters known to be associated with thyroid function differed between the thyrotoxicosis and control groups. Many studies have reported that the thyrotoxic condition decreases serum creatinine levels [21,22] and improves lipid profiles, including total cholesterol [23,24]. Thyrotoxicosis increases liver enzyme levels owing to relative hypoxia in the hepatic perivenular regions [25,26]. Although reports have conflicted, relatively low platelet counts in thyrotoxic patients have also been reported [27,28]. Our baseline results were consistent with these previous reports.

HSS, on-site rHR, WD-rHR-own, and WD-rHR-Fitbit were decreased as thyrotoxicosis improved and these variables were all associated with serum free T4 levels and thyrotoxicosis in linear and binary logistic model GEE analyses, respectively. Interestingly, standardized on-site rHR showed relatively lower beta for serum free T4 level and a lower OR for thyrotoxicosis compared with other standardized parameters in the GEE analyses. On-site rHR is a one-time measurement of one clinical aspect, while HSS is a one-time measurement of several clinical aspects and WD-rHR is calculated from continuously monitored data for one clinical aspect. These differences can explain on-site rHR’s relatively weak correlation with thyroid function compared with HSS or WD-rHR. Similar beta and ORs and their 95% CIs between standardized HSS and WD-rHR suggest that WD-rHR calculated from continuously collected, detailed data by wearable devices may be used to assess thyroid function status with similar accuracy and precision with HSS, a validated assessment tool known to reflect thyroid function, but which also requires time and an educated scorer [29,30]. Considering the time and effort required for evaluating HSS, WD-rHR, a simple parameter based on a large volume of detailed HR and activity data, could be a better predictor because the patient could get the results only by wearing the device. Even if patients measure their rHR manually between clinic visits, they still need to maintain a resting position without activity for 10 to 15 minutes. This makes it callenging to measure rHR manually several times daily. Taken together, WD-rHR is not only as accurate as a multi-dimensional assessment tool developed for evaluating thyrotoxic status, but also easier to measure compared with other assessment tools or conventional methods of measuring rHR.

Current wearable devices allow highly detailed, longitudinal measurement of physical indices. This large volume of clinical information (ie, “high definition data”) may provide more accurate and objective information than subjective symptoms or physical signs recorded at clinic visits, which can be influenced by diurnal variation, emotional state, and other factors [31]. In this study, we calculated rHR from continuously monitored HR and physical activity data. Similar to manually measured HR, HR measured by wearable devices can also be affected by various factors, such as body position or emotional state. This influence cannot be selectively removed in the process of extracting rHR from collected HR data because these factors cannot be detected by the device we used. With the algorithm we used to calculate rHR, we could remove only the influence of physical activity, which can be detected by the device. However, continuously monitored high definition data could blunt the influence of other factors. As mentioned above, we selected rHR as a median HR value from data collected within a given time window without physical activity during the day. This algorithm might minimize the effect of daily repeated short-term factors such as emotional state or body position. However, long-term and continuous factors, such as disease status, still influence the data. Therefore, WD-rHR-own might reflect the disease state of thyrotoxicosis more accurately than on-site rHR. Although the algorithm for extracting resting HR provided by Fitbit is proprietary and undisclosed, the basic concept is not different from ours, and WD-rHR-Fitbit showed a similar association with thyroid function compared with WD-rHR-own. Based on the results of the present study, we suggest that WD-rHR shows reasonable a prediction of thyroid function in thyrotoxic patients. Also, the development of a wearable device capable of measuring other parameters such as sweating, tremor, or respiration rate would further improve accurate prediction of thyroid function status. Therefore, “high-definition medicine”, through wearable devices, shows current capability and future potential in its clinical application in this field.

This study has some significant clinical implications. Although monitoring thyroid function using wearable devices cannot replace TFT, it could aid the management of thyrotoxicosis. During the treatment of GD, most patients are expected to repeat the TFT every one to two months regardless of their response to ATDs, and the interventions including dose adjustment of ATDs are provided with the same time interval. Monitoring thyroid function using wearable devices may provide patients with individualized, flexible, and more accurate interventions during treatment and follow-up, minimizing inconvenience and costs. Moreover, about 50% of patients have been reported to relapse within two years after discontinuing ATD, even if they were treated according to the recommended guidelines [32]. Monitoring during everyday life using wearable devices may enable early detection of disease recurrence in patients who are in remission after treatment. Although further studies are needed, these results may be applied to the monitoring of drug-induced subclinical thyrotoxicosis in patients with differentiated thyroid cancer (DTC) who are undergoing TSH-suppressive therapy (ie, maintaining iatrogenic subclinical thyrotoxic status using thyroid hormone over-replacement). TSH suppressive therapy is recommended to prevent the growth of DTC [33], but over-suppression of TSH increases risks of fracture and cardiovascular disease [34-36]. Therefore, a simple monitoring tool during TSH-suppressive therapy, such as a wearable device in everyday life, might help maintain appropriate intensities of TSH suppression. We have developed a web application to predict thyrotoxicosis using rHR data from wearable devices [37] based on the results of this study. Since the present study was a small, observational, preliminary study, additional studies are planned to investigate the clinical usefulness of this application for thyrotoxic patients and DTC patients undergoing TSH suppressive therapy.

The primary strength of the study was that it contributes to the literature by monitoring HR throughout the day using wearable devices, for the first time, in patients with thyrotoxicosis. The clinical evidence provided herein may inspire further investigations and clinical applications of biosignals monitored by wearable devices in thyroid dysfunction. The other strength is that we used commercially available wearable devices and mobile applications. Thus, our results can be immediately applied to the management of thyrotoxic patients and provide a ready-to-use algorithm for making Web-based or mobile applications for managing thyrotoxicosis.

This study also has some limitations, which should be considered when interpreting the results. First, it was likely easier for younger people, who are adept at using mobile phones and wearable devices, to participate in the study, although the participants’ ranged from 18 to 60 years of age. Also, signs such as increased HR owing to thyrotoxicosis may not be as evident in the elderly [5]. Therefore, it may be difficult to apply the results of this study to those aged over 65 years. However, the ages of the participants in this study were similar to the population in which thyrotoxicosis is most prevalent, and the results of this study can likely be applied to those who can use mobile phones and wearable devices. Second, this study has a small sample size and imbalance between patient and control group sizes, as well as the single-center based design. Among the 30 thyrotoxic patients, only 3 patients were diagnosed as thyroiditis, and we could not perform statistical analyses in these three patients separately. We analyzed the patients with GD only, and the results showed the same tendency as those in all patients (Multimedia Appendix 1). Despite the imbalance between patient and control group sizes, all statistical powers of the analyses in Table 1 and 2 were over 90%. Further investigations, including more participants and study sites, are needed to confirm the results of this study.

### Conclusion

In conclusion, our results indicate that rHR data from wearable devices show reasonable predictability of thyroid function in patients with thyrotoxicosis. This parameter can be measured relatively simply and may be useful as well as clinically feasible for the management of thyroid dysfunction. This study is a starting point for the clinical application of high-definition medicine in the management of thyroid disease.

## Appendix

Multimedia Appendix 1 Inclusion and exclusion criteria, biochemical measurements, and generalized estimating equations analyses.

## Abbreviations

ANOVA: analysis of variance
ATD: antithyroid drug
CRF: case report form
DTC: differentiated thyroid cancer
GD: Graves’ disease
GEE: generalized estimating equation
HR: heart rate
HSS: Hyperthyroidism Symptom Scale
IQR: interquartile range
JSON: JavaScript object notation
OR: odds ratio
SNUBH: Seoul National University Bundang Hospital
T3: triiodothyronine
T4: thyroxine
TFT: thyroid function test
TSH: thyroid hormone stimulating hormone
WD-rHR-Fitbit: resting heart rate from wearable device derived by Fitbit algorithm
WD-rHR-own: resting heart rate from wearable device derived by own algorithm
