# Supplementary material for: Clinical Feasibility of Monitoring Resting Heart Rate Using a Wearable Activity Tracker in Patients With Thyrotoxicosis: Prospective Longitudinal Observational Study
Source: JMIR Mhealth Uhealth. 2018 Jul 13;6(7):e159. doi: 10.2196/mhealth.9884 (PMC6064040; doi:10.2196/mhealth.9884)
Supplement: Multimedia Appendix 1 [file mhealth_v6i7e159_app1.pdf]

Supplementary online to:

Clinical feasibility of monitoring resting heart rate using a wearable activity tracker in patients with thyrotoxicosis: prospective longitudinal observational study

Jie-Eun Lee[1], Dong Hwa Lee[2], Tae Jung Oh[2], Kyoung Min Kim[2], Sung Hee Choi[2], Soo Lim[2], Young Joo Park[3], Do Joon Park[3], Hak Chul Jang [2], Jae Hoon Moon[2]

Supplementary contents:

1. Supplement Table 1
2. eMethods
3. Supplement Table 2
4. Supplement Table 3
5. Supplement Table 4
6. Supplement Table 5

Supplementary methods

Supplement Table 1. Inclusion and exclusion criteria for thyrotoxicosis and control groups

Group	Inclusion criteria	Exclusion criteria
Patients	1) Aged 15–60 years<br>2) Diagnosed with newly developed or recurrent thyrotoxicosis<br>3) Who can use a wearable device and smartphone apps<br>4) Planned to be treated with ATDs if affected by Graves' disease	1) A history of thyrotoxic periodic paralysis<br>2) Thyrotoxicosis caused by toxic nodular goiter<br>3) Taking medications that can affect heart rate (except short-acting beta-blockers prescribed to relieve thyrotoxic symptoms)
Controls	1) Aged 15–60 years<br>2) Confirmed euthyroid state by thyroid function test (TFT)<br>3) Who can use a wearable device and smartphone apps	1) A history of thyroid disease or taking thyroid hormone or an ATD<br>2) Taking medications that can affect heart rate

Supplement Methods. Biochemical Measurements

Serum levels of blood urea nitrogen (BUN), creatinine, and glucose were measured by automated standard laboratory methods (Hitachi 747; Hitachi, Tokyo, Japan). Serum total protein, albumin, total bilirubin, aspartate aminotransferase (AST), and alanine aminotransferase (ALT) were measured with an autoanalyzer (TBA-200FR; Toshiba, Tokyo, Japan). Complete blood count was performed on a Coulter AC.T diff2 Analyzer (Beckman Coulter, Brea, CA, USA). For the TFT, concentrations of serum free T4 (DiaSorin, Saluggia, Italy) and thyroid-stimulating hormone (TSH; CIS Bio International, Gif-sur-Yvette, France) were measured using immunoradiometric assays.

Supplementary Table 2. Generalized estimating equations analyses (linear model) for the association between free T4 and associating parameters in thyrotoxic patients

	B	95% CI	p
Beta-blocker users (n = 13)			
Hyperthyroid Symptom Scale	0.738	0.456 - 1.021	< 0.001
On-site rHR	0.542	0.132 - 0.953	0.010
WD-rHR-own	0.567	0.274 - 0.860	< 0.001
WD-rHR-fitbit	0.609	0.312 - 0.907	< 0.001
Non-beta-blocker users (n = 15)			
Hyperthyroid Symptom Scale	0.244	0.104 - 0.385	0.001
On-site rHR	0.174	0.012 - 0.336	0.036
WD-rHR-own	0.304	0.133 - 0.476	< 0.001
WD-rHR-fitbit	0.307	0.098 - 0.516	0.004

Parameters are standardized to have the same mean and SD (mean = 0 and SD = 1.0) for comparison and analyzed separately. CI, confidence interval; rHR, resting heart rate; WD-rHR-own, rHR from wearable device derived by own algorithm; -fitbit, derived by Fitbit algorithm.

Supplementary Table 3. Generalized estimating equations analyses (binary logistic model) for the association between thyrotoxicosis and associating parameters in thyrotoxic patients

	OR	95% CI	*p*
Beta-blocker users (n = 13)			
Hyperthyroid Symptom Scale	2.600	1.179 - 5.733	0.018
On-site rHR	1.744	1.024 - 2.969	0.041
WD-rHR-own	2.160	1.235 - 3.779	0.007
WD-rHR-fitbit	2.752	1.373 - 5.515	0.004
Non-beta-blocker users (n = 15)			
Hyperthyroid Symptom Scale	2.701	1.607 - 4.539	< 0.001
On-site rHR	1.559	0.769 - 3.164	0.218
WD-rHR-own	2.807	1.154 - 6.824	0.023
WD-rHR-fitbit	3.182	1.248 - 8.110	0.015

Parameters are standardized to have the same mean and SD (mean = 0 and SD = 1.0) for comparison and analyzed separately. OR, odds ratio; CI, confidence interval; rHR, resting heart rate; WD-rHR-own, rHR from wearable device derived by own algorithm; -fitbit, derived by Fitbit algorithm.

Supplementary Table 4. Generalized estimating equations analyses (linear model) for the association between free T4 and associating parameters in patients with Graves' disease (n = 25)

	B	95% CI	p
Hyperthyroid Symptom Scale	0.495	0.311 - 0.679	< 0.001
On-site rHR	0.396	0.146 - 0.645	0.002
WD-rHR-own	0.471	0.293 - 0.648	< 0.001
WD-rHR-fitbit	0.503	0.318 - 0.689	< 0.001

Parameters are standardized to have same mean and SD (mean = 0 and SD = 1.0) for comparison and analyzed separately. CI, confidence interval; rHR, resting heart rate; WD-rHR-own, rHR from wearable device derived by own algorithm; -fitbit, derived by Fitbit algorithm.

Supplementary Table 5. Generalized estimating equations analyses (binary logistic model) for the association between thyrotoxicosis and associating parameters in patients with Graves' disease (n = 25)

	OR	95% CI	*p*
Hyperthyroid Symptom Scale	2.430	1.481 - 3.987	< 0.001
On-site rHR	1.799	1.101 - 2.940	0.019
WD-rHR-own	2.274	1.406 - 3.678	0.001
WD-rHR-fitbit	2.589	1.580 - 4.242	< 0.001

Parameters are standardized to have same mean and SD (mean = 0 and SD = 1.0) for comparison and analyzed separately. OR, odds ratio; CI, confidence interval; rHR, resting heart rate; WD-rHR-own, rHR from wearable device derived by own algorithm; -fitbit, derived by Fitbit algorithm.
